# Supplementary material for: Preoperative Brain Tumor Imaging: Models and Software for Segmentation and Standardized Reporting
Source: Front Neurol. 2022 Jul 27;13:932219. doi: 10.3389/fneur.2022.932219 (PMC9364874; doi:10.3389/fneur.2022.932219)
Supplement: Supplementary file 1 [file Data_Sheet_1.pdf]

Supplementary Material

1 METRICS

Given a ground truth segmentation (denoted as g) and an automatic segmentation coming from a trained model (denoted as d), the following four voxel-wise cardinalities can be defined, given a total amount of voxels X in a given MRI volume (of dimensions $H \times W \times D$).

$$TP = \sum_{x=1}^{|X|} (g(x) == 1) \ \ \& \ \ (d(x) == 1) \tag{S1}$$

$$TN = \sum_{x=1}^{|X|} (g(x) == 0) \ \ \& \ \ (d(x) == 0) \tag{S2}$$

$$FP = \sum_{x=1}^{|X|} (g(x) == 0) \ \ \& \ \ (d(x) == 1) \tag{S3}$$

$$FN = \sum_{x=1}^{|X|} (g(x) == 1) \ \ \& \ \ (d(x) == 0) \tag{S4}$$

Using those cardinalities as base elements, the other voxel-wise metrics can be computed according to the following equations.

True Positive Rate (TPR):

$$TPR = \frac{TP}{TP + FN} \tag{S5}$$

True Negative Rate (TNR):

$$TNR = \frac{TN}{TN + FP} \tag{S6}$$

False Positive Rate (FPR):

$$FPR = \frac{FP}{FP + TN} \tag{S7}$$

False Negative Rate (FNR):

$$FNR = \frac{FN}{FN + TP} \tag{S8}$$

Positive Predictive Value (PPV):

$$PPV = \frac{TP}{TP + FP} \tag{S9}$$

Dice:

$$Dice = \frac{2 \cdot TP}{2 \cdot TP + FP + FN} \tag{S10}$$

Jaccard:

$$J = \frac{TP}{TP + FP + FN} \tag{S11}$$

Intersection over Union (IoU):

$$IoU = \frac{\sum_{x=1}^{|X|} g(x) \cap d(x)}{\sum_{x=1}^{|X|} (g(x) \cup d(x))} \tag{S12}$$

Area Under the Curve (AUC):

$$AUC = 1 - \frac{FPR + FNR}{2} \tag{S13}$$

Global Consistency Error (GCE):

$$GCE = \frac{1}{X} min\left\{ \frac{FN(FN + 2TP)}{TP + FN} + \frac{FP(FP + 2TN)}{TN + FP}, \frac{FP(FP + 2TP)}{TP + FP} + \frac{FN(FN + 2TN)}{TN + FN} \right\} \tag{S14}$$

Mathews Correlation Coefficient (MCC):

$$MCC = \frac{(TP \cdot TN) - (FP \cdot FN)}{\sqrt{(TP + FP) \cdot (TP + FN) \cdot (TN + FP) \cdot (TN + FN)}} \tag{S15}$$

Adjusted Rand Index (ARI):

$$\begin{aligned} a = 0.5 \cdot ((TP \cdot (TP - 1)) + (FP \cdot (FP - 1)) + (TN \cdot (TN - 1)) + (FN \cdot (FN - 1))) \\ b = 0.5 \cdot (((TP + FN)^2 + (TN + FP)^2) - (TP^2 + TN^2 + FP^2 + FN^2)) \\ c = 0.5 \cdot (((TP + FP)^2 + (TN + FN)^2) - (TP^2 + TN^2 + FP^2 + FN^2)) \\ d = \frac{(H \cdot W \cdot D) \cdot (H \cdot W \cdot D - 1)}{2 \cdot (a + b + c)} \\ ARI = \frac{2 \cdot (a \cdot b - b \cdot c)}{c^2 + b^2 + 2 \cdot a \cdot b + (a + d) \cdot (c + b)} \end{aligned} \tag{S16}$$

Variation Of Information (VOI):

$$\begin{aligned}
h_1 &= -\left(\frac{FN+TP}{X} \cdot log_2\left(\frac{FN+TP}{X}\right) + \frac{1-(FN+TP)}{X} \cdot log_2\left(\frac{1-(FN+TP)}{X}\right)\right) \\
h_2 &= -\left(\frac{FP+TP}{X} \cdot log_2\left(\frac{FP+TP}{X}\right) + \frac{1-(FP+TP)}{X} \cdot log_2\left(\frac{1-(FP+TP)}{X}\right)\right) \\
p_{00} &= 1 \; if \; TN == 0 \; else \; \frac{TN}{X} \\
p_{01} &= 1 \; if \; FN == 0 \; else \; \frac{FN}{X} \\
p_{10} &= 1 \; if \; FP == 0 \; else \; \frac{FP}{X} \\
p_{11} &= 1 \; if \; TP == 0 \; else \; \frac{TP}{X} \\
h_{12} &= -\left(log_2(p_{00}) \cdot \frac{TN}{X} + log_2(p_{01}) \cdot \frac{FN}{X} + log_2(p_{10}) \cdot \frac{FP}{X} + log_2(p_{11}) \cdot \frac{TP}{X}\right) \\
MI &= h_1 + h_2 - h_{12} \\
VOI &= h_1 + h_2 - (2 \cdot MI)
\end{aligned} \tag{S17}$$

Probabilistic Distance (PBD):

$$PBD = \frac{\sum_{x=1}^{|X|} |g(x) - d(x)|}{2 \cdot \sum_{x=1}^{|X|} (g(x) \cdot d(x))} \tag{S18}$$

2 METRICS ANALYSIS

For each of the four brain tumor subtypes, the following elements are consistently reported: the fold-wise overall performances, the metrics correlation matrix, and a figure showing outlier cases according to different metrics.

2.1 Glioblastoma segmentation

Overall segmentation performances for the glioblastoma tumor type are reported fold-wise in Table S1. The average Dice score is the lowest on fold 9 which is unsurprising given the size of the test set, the largest of all. Otherwise, the average Dice score is relatively stable across all folds between 85% and 88%.

The correlation matrix between some of the considered validation metrics is shown in Table S2. Visual segmentation results are illustrated in Fig. S1 where the GCE, PBD, RAVD, and HD95 metrics scored orders of magnitude above their average, indicating clear outliers not identifiable by the Dice metric only. In the first row, the two leftmost examples exhibit undersegmentation and oversegmentation of the tumor. The two rightmost examples are indicating subpar manual ground truth and where predictions from the model appears slightly more accurate. In the second row, the two leftmost tumors where almost completely missed by the model, while only the contrast-enhancing part of the tumor was segmented in the two rightmost cases. In the third row, the third patient features both a glioblastoma and a meningioma. Unfortunately, the trained model is not meant to perform tumor type classification and both tumor types are visually quite similar. As a result, both tumors have been segmented hence the low metric scores but for a 94.69% Dice over the sole glioblastoma. In the first and last patient, the cyst does not seem to have been included in the ground truth, focusing only on the contrast-enhanced parts. Finally in the fourth row, all outlier cases featured a tumor with a very small volume whereby the HD95 scored low while all Dice scores reached above 80%.

Table S1. Fold-wise overall segmentation performance for the glioblastoma tumor type.

		Pixel-wise		Patient-wise			Object-wise		
Fold	# Samples	Dice	Dice-TP	F1-score	Recall	Precision	F1-score	Recall	Precision
0	153	87.97 ± 12.28	88.55 ± 10.03	99.01	99.35	98.68	93.27	89.27	97.64
1	103	88.54 ± 13.95	90.29 ± 06.35	98.19	98.06	98.32	92.97	90.58	95.49
2	75	86.44 ± 16.05	86.44 ± 16.05	98.75	100.00	97.52	94.44	92.66	96.30
3	72	86.17 ± 15.70	87.39 ± 12.00	97.17	98.61	95.77	90.59	89.80	91.40
4	293	87.03 ± 15.21	89.16 ± 06.87	96.54	97.61	95.50	90.28	89.30	91.28
5	83	88.22 ± 10.79	88.22 ± 10.79	98.57	100.00	97.19	89.88	85.50	94.74
6	38	84.73 ± 17.91	84.73 ± 17.91	99.34	100.00	98.68	80.34	68.06	98.04
7	74	83.49 ± 19.42	87.01 ± 09.36	96.99	95.89	98.12	96.39	96.39	96.39
8	49	86.84 ± 15.89	88.65 ± 09.87	98.46	97.96	98.96	83.67	72.73	98.48
9	457	82.82 ± 21.85	85.25 ± 16.88	96.10	97.16	95.07	86.87	82.44	91.82
10	249	84.70 ± 20.42	87.88 ± 12.38	97.08	96.39	97.79	88.36	81.57	96.37
11	134	86.17 ± 15.45	87.47 ± 11.32	98.87	98.50	99.24	86.30	76.61	98.81
12	86	86.80 ± 12.66	87.82 ± 08.51	97.44	98.84	96.08	92.05	90.91	93.22
13	171	85.12 ± 11.19	85.62 ± 09.11	97.17	99.42	95.02	92.68	94.67	90.76
14	97	88.77 ± 10.38	88.77 ± 10.38	99.13	100.00	98.28	88.15	80.86	96.88

Table S2. Metrics correlation matrix for the glioblastoma segmentation. The color intensity of each cell represents the strength of the correlation, where blue denotes direct correlation and red denotes inverse correlation.

	Overlap						Volume		Information theory		Probabilistic					Spatial distance			Instance-wise	
	Dice	TPR	TNR	PPV	IOU	GCE	VS	RAVD	MI	VOI	CKS	AUC	VC	MCC	PBD	HD95	MHD	ASSD	ARI	OASSD
Dice	1.0	0.7	0.29	0.62	0.98	-0.22	0.94	-0.35	0.99	-0.23	1.0	0.71	0.78	1.0	-0.34	-0.55	-0.43	-0.71	1.0	-0.3
TPR	0.7	1.0	-0.17	-0.07	0.71	-0.08	0.62	0.1	0.7	-0.08	0.7	1.0	0.51	0.71	-0.26	-0.38	-0.34	-0.47	0.7	-0.2
TNR	0.29	-0.17	1.0	0.58	0.28	-0.76	0.29	-0.36	0.33	-0.76	0.29	-0.17	0.23	0.29	-0.04	-0.16	-0.04	-0.27	0.29	-0.22
PPV	0.62	-0.07	0.58	1.0	0.64	-0.24	0.55	-0.49	0.64	-0.25	0.62	-0.07	0.47	0.63	-0.16	-0.38	-0.21	-0.47	0.62	-0.22
IOU	0.98	0.71	0.28	0.64	1.0	-0.24	0.9	-0.29	0.99	-0.24	0.98	0.71	0.71	0.99	-0.28	-0.55	-0.37	-0.7	0.98	-0.31
GCE	-0.22	-0.08	-0.76	-0.24	-0.24	1.0	-0.19	0.13	-0.3	1.0	-0.23	-0.09	-0.14	-0.23	0.02	0.18	0.03	0.29	-0.23	0.28
VS	0.94	0.62	0.29	0.55	0.9	-0.19	1.0	-0.37	0.9	-0.2	0.94	0.62	0.76	0.92	-0.36	-0.48	-0.43	-0.65	0.94	-0.26
RAVD	-0.35	0.1	-0.36	-0.49	-0.29	0.13	-0.37	1.0	-0.31	0.15	-0.35	0.1	-0.39	-0.34	0.18	0.19	0.14	0.28	-0.35	0.15
MI	0.99	0.7	0.33	0.64	0.99	-0.3	0.9	-0.31	1.0	-0.31	0.99	0.7	0.74	0.99	-0.31	-0.56	-0.4	-0.71	0.99	-0.32
VOI	-0.23	-0.08	-0.76	-0.25	-0.24	1.0	-0.2	0.15	-0.31	1.0	-0.23	-0.08	-0.15	-0.24	0.03	0.18	0.03	0.3	-0.24	0.28
CKS	1.0	0.7	0.29	0.62	0.98	-0.23	0.94	-0.35	0.99	-0.23	1.0	0.71	0.78	1.0	-0.34	-0.55	-0.43	-0.71	1.0	-0.3
AUC	0.71	1.0	-0.17	-0.07	0.71	-0.09	0.62	0.1	0.7	-0.08	0.71	1.0	0.51	0.71	-0.27	-0.38	-0.34	-0.47	0.71	-0.2
VC	0.78	0.51	0.23	0.47	0.71	-0.14	0.76	-0.39	0.74	-0.15	0.78	0.51	1.0	0.78	-0.49	-0.51	-0.58	-0.71	0.78	-0.22
MCC	1.0	0.71	0.29	0.63	0.99	-0.23	0.92	-0.34	0.99	-0.24	1.0	0.71	0.78	1.0	-0.36	-0.55	-0.44	-0.71	1.0	-0.31
PBD	-0.34	-0.26	-0.04	-0.16	-0.28	0.02	-0.36	0.18	-0.31	0.03	-0.34	-0.27	-0.49	-0.36	1.0	0.16	0.97	0.29	-0.34	0.05
HD95	-0.55	-0.38	-0.16	-0.38	-0.55	0.18	-0.48	0.19	-0.56	0.18	-0.55	-0.38	-0.51	-0.55	0.16	1.0	0.25	0.89	-0.55	0.14
MHD	-0.43	-0.34	-0.04	-0.21	-0.37	0.03	-0.43	0.14	-0.4	0.03	-0.43	-0.34	-0.58	-0.44	0.97	0.25	1.0	0.4	-0.43	0.06
ASSD	-0.71	-0.47	-0.27	-0.47	-0.7	0.29	-0.65	0.28	-0.71	0.3	-0.71	-0.47	-0.71	-0.71	0.29	0.89	0.4	1.0	-0.71	0.2
ARI	1.0	0.7	0.29	0.62	0.98	-0.23	0.94	-0.35	0.99	-0.24	1.0	0.71	0.78	1.0	-0.34	-0.55	-0.43	-0.71	1.0	-0.3
OASSD	-0.3	-0.2	-0.22	-0.22	-0.31	0.28	-0.26	0.15	-0.32	0.28	-0.3	-0.2	-0.22	-0.31	0.05	0.14	0.06	0.2	-0.3	1.0

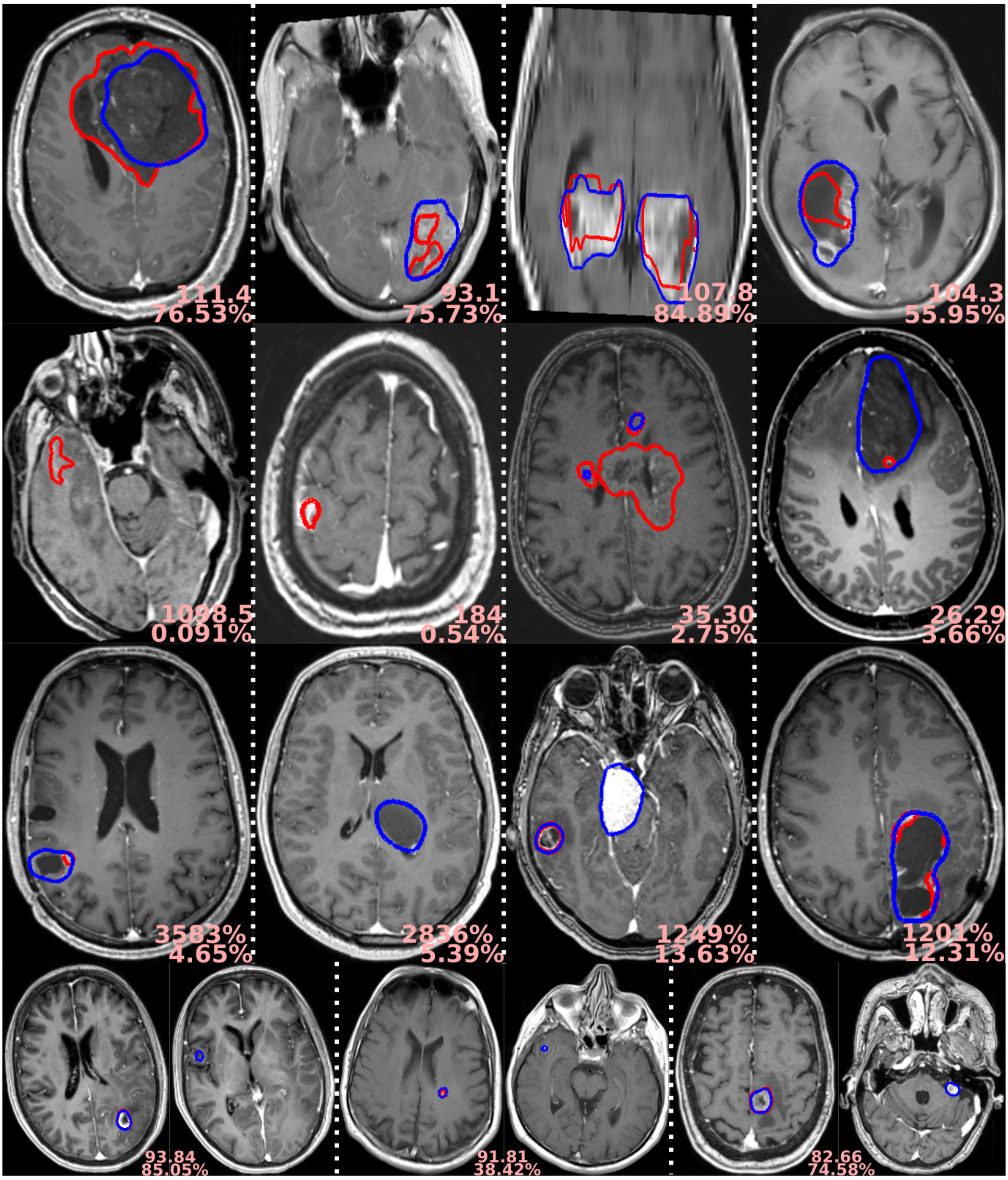

Figure S1. Examples of glioblastoma outliers where the ground truth is shown in red and the automatic segmentation in blue. Each patient is separated by a dotted white line, and each row focuses on a different metric, from top to bottom: GCE, PBD, RAVD, and HD95. For each patient, both metric of interest and Dice scores are provided in light red.

2.2 Lower grade glioma segmentation

Overall segmentation performances for the lower grade glioma tumor type are reported fold-wise in Table S3. The average Dice score is quite stable across the different folds, at 75%, given the almost identical number of samples they hold. The same trend can be noticed for the patient-wise and object-wise metrics across the board.

The correlation matrix between some of the considered validation metrics is shown in Table S4. Visual segmentation results are illustrated in Fig. S2 where the GCE, PBD, RAVD, and HD95 metrics scored orders of magnitude above their average, indicating clear outliers not identifiable by the Dice metric only. In the first row, all examples exhibit extremely large and diffuse tumors whereby the manual ground truth is far from smooth and often parcelled. While Dice scores are overall above 60%, one can notice that only the brighter regions were segmented by the model. In the second row, tumors with a small volume were not segmented while a bright non-tumor region has been segmented in the third case. In the last row, small regions far from the main tumor location were incorrectly segmented, leading to high HD95 values for reasonable to good Dice scores.

Table S3. Fold-wise overall performance for the lower grade glioma segmentation.

		Pixel-wise		Patient-wise			Object-wise		
Fold	# Samples	Dice	Dice-TP	F1-score	Recall	Precision	F1-score	Recall	Precision
0	132	74.97 ± 28.56	83.87 ± 12.90	91.09	89.39	92.85	78.23	72.78	84.56
1	132	77.46 ± 22.83	80.50 ± 17.21	94.86	96.21	93.55	80.59	76.47	85.19
2	132	74.98 ± 25.20	79.18 ± 18.40	94.68	94.70	94.67	83.68	77.78	90.54
3	130	76.54 ± 22.71	80.24 ± 15.61	95.44	95.38	95.49	80.94	72.63	91.39
4	132	73.01 ± 29.77	82.36 ± 15.16	91.98	88.64	95.58	84.47	78.18	91.85

Table S4. Metrics correlation matrix for the lower grade glioma segmentation. The color intensity of each cell represents the strength of the correlation, where blue denotes direct correlation and red denotes inverse correlation.

	Overlap						Volume		Information theory		Probabilistic					Spatial distance			Instance-wise	
	Dice	TPR	TNR	PPV	IOU	GCE	VS	RAVD	MI	VOI	CKS	AUC	VC	MCC	PBD	HD95	MHD	ASSD	ARI	OASSD
Dice	1.0	0.83	0.15	0.48	0.98	-0.2	0.91	-0.11	0.98	-0.2	1.0	0.83	0.81	1.0	-0.45	-0.51	-0.66	-0.67	1.0	-0.34
TPR	0.83	1.0	-0.15	-0.02	0.8	-0.16	0.74	0.28	0.8	-0.15	0.83	1.0	0.67	0.82	-0.4	-0.37	-0.56	-0.51	0.83	-0.29
TNR	0.15	-0.15	1.0	0.49	0.19	-0.66	0.12	-0.34	0.24	-0.68	0.16	-0.14	0.01	0.16	0.03	-0.14	-0.0	-0.15	0.16	-0.21
PPV	0.48	-0.02	0.49	1.0	0.54	-0.17	0.32	-0.65	0.54	-0.19	0.48	-0.02	0.36	0.51	-0.01	-0.41	-0.19	-0.45	0.48	-0.23
IOU	0.98	0.8	0.19	0.54	1.0	-0.24	0.87	-0.11	0.99	-0.24	0.98	0.8	0.74	0.98	-0.37	-0.53	-0.59	-0.66	0.98	-0.36
GCE	-0.2	-0.16	-0.66	-0.17	-0.24	1.0	-0.15	0.02	-0.3	0.99	-0.21	-0.16	-0.04	-0.21	-0.02	0.18	0.01	0.24	-0.22	0.32
VS	0.91	0.74	0.12	0.32	0.87	-0.15	1.0	-0.1	0.86	-0.16	0.91	0.74	0.75	0.89	-0.49	-0.38	-0.63	-0.58	0.91	-0.25
RAVD	-0.11	0.28	-0.34	-0.65	-0.11	0.02	-0.1	1.0	-0.12	0.04	-0.11	0.28	-0.1	-0.12	0.0	0.11	0.02	0.12	-0.11	0.02
MI	0.98	0.8	0.24	0.54	0.99	-0.3	0.86	-0.12	1.0	-0.31	0.99	0.81	0.76	0.99	-0.4	-0.54	-0.61	-0.68	0.99	-0.38
VOI	-0.2	-0.15	-0.68	-0.19	-0.24	0.99	-0.16	0.04	-0.31	1.0	-0.21	-0.16	-0.04	-0.21	-0.02	0.19	0.01	0.25	-0.22	0.33
CKS	1.0	0.83	0.16	0.48	0.98	-0.21	0.91	-0.11	0.99	-0.21	1.0	0.83	0.81	1.0	-0.45	-0.51	-0.66	-0.67	1.0	-0.35
AUC	0.83	1.0	-0.14	-0.02	0.8	-0.16	0.74	0.28	0.81	-0.16	0.83	1.0	0.67	0.83	-0.4	-0.37	-0.56	-0.51	0.83	-0.29
VC	0.81	0.67	0.01	0.36	0.74	-0.04	0.75	-0.1	0.76	-0.04	0.81	0.67	1.0	0.81	-0.51	-0.52	-0.73	-0.72	0.81	-0.24
MCC	1.0	0.82	0.16	0.51	0.98	-0.21	0.89	-0.12	0.99	-0.21	1.0	0.83	0.81	1.0	-0.46	-0.52	-0.67	-0.68	1.0	-0.36
PBD	-0.45	-0.4	0.03	-0.01	-0.37	-0.02	-0.49	0.0	-0.4	-0.02	-0.45	-0.4	-0.51	-0.46	1.0	0.16	0.87	0.26	-0.45	0.1
HD95	-0.51	-0.37	-0.14	-0.41	-0.53	0.18	-0.38	0.11	-0.54	0.19	-0.51	-0.37	-0.52	-0.52	0.16	1.0	0.37	0.84	-0.51	0.16
MHD	-0.66	-0.56	-0.0	-0.19	-0.59	0.01	-0.63	0.02	-0.61	0.01	-0.66	-0.56	-0.73	-0.67	0.87	0.37	1.0	0.45	-0.66	0.16
ASSD	-0.67	-0.51	-0.15	-0.45	-0.66	0.24	-0.58	0.12	-0.68	0.25	-0.67	-0.51	-0.72	-0.68	0.26	0.84	0.45	1.0	-0.67	0.23
ARI	1.0	0.83	0.16	0.48	0.98	-0.22	0.91	-0.11	0.99	-0.22	1.0	0.83	0.81	1.0	-0.45	-0.51	-0.66	-0.67	1.0	-0.35
OASSD	-0.34	-0.29	-0.21	-0.23	-0.36	0.32	-0.25	0.02	-0.38	0.33	-0.35	-0.29	-0.24	-0.36	0.1	0.16	0.16	0.23	-0.35	1.0

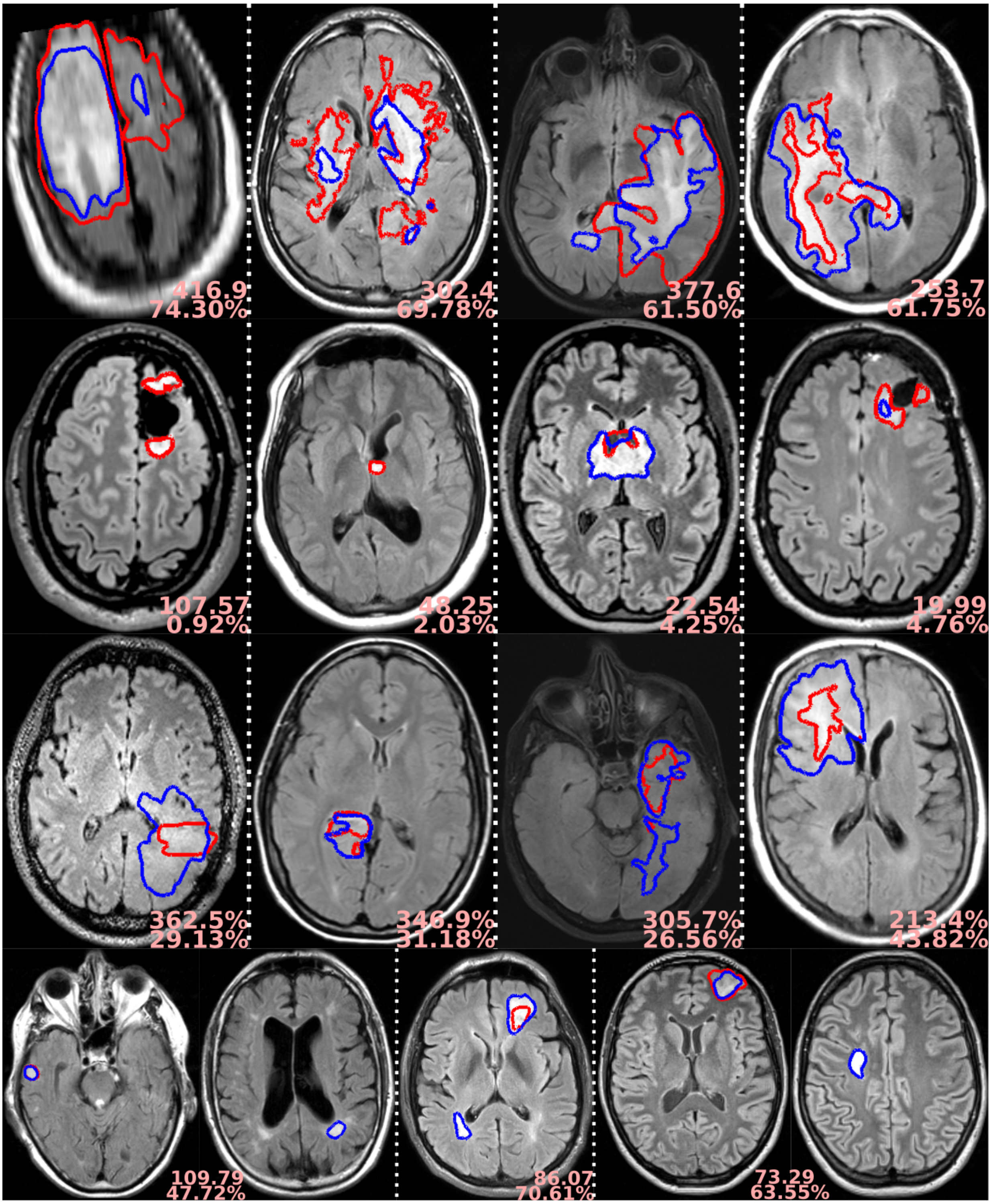

Figure S2. Examples of lower grade glioma outliers where the ground truth is shown in red and the automatic segmentation in blue. Each patient is separated by a dotted white line, and each row focuses on a different metric, from top to bottom: GCE, PBD, RAVD, and HD95. For each patient, both metric of interest and Dice scores are provided in light red.

2.3 Meningioma segmentation

Overall segmentation performances for the meningioma tumor type are reported fold-wise in Table S5. The average Dice score is quite stable across the different folds, at 75%, given the almost identical number of samples they hold. The same trend can be noticed for the patient-wise and object-wise metrics across the board.

The correlation matrix between some of the considered validation metrics is shown in Table S6. Visual segmentation results are illustrated in Fig. S3 where the GCE, PBD, RAVD, and HD95 metrics scored orders of magnitude above their average, indicating clear outliers not identifiable by the Dice metric only. Coronal slices are provided in case of a large slice thickness.

Table S5. Fold-wise overall performance for the meningioma segmentation.

		Pixel-wise		Patient-wise			Object-wise		
Fold	# Samples	Dice	Dice-TP	F1-score	Recall	Precision	F1-score	Recall	Precision
0	144	77.30 ± 28.18	84.32 ± 16.55	87.88	91.67	84.39	77.60	83.33	72.60
1	144	74.93 ± 31.61	85.63 ± 15.02	91.83	87.50	96.60	83.08	75.00	93.10
2	144	74.94 ± 29.94	83.65 ± 16.50	91.23	89.58	92.94	87.58	87.82	87.34
3	143	74.76 ± 32.53	87.62 ± 10.66	91.20	85.31	97.97	87.20	79.25	96.92
4	144	73.07 ± 30.46	82.85 ± 15.57	91.19	88.19	94.40	83.82	79.23	88.96

Table S6. Metrics correlation matrix for the meningioma segmentation. The color intensity of each cell represents the strength of the correlation, where blue denotes direct correlation and red denotes inverse correlation.

	Overlap						Volume		Information theory		Probabilistic					Spatial distance			Instance-wise	
	Dice	TPR	TNR	PPV	IOU	GCE	VS	RAVD	MI	VOI	CKS	AUC	VC	MCC	PBD	HD95	MHD	ASSD	ARI	OASSD
Dice	1.0	0.85	0.11	0.4	0.98	-0.34	0.94	-0.04	0.99	-0.35	1.0	0.85	0.82	1.0	-0.29	-0.43	-0.68	-0.56	1.0	-0.28
TPR	0.85	1.0	-0.13	-0.07	0.84	-0.34	0.77	0.4	0.85	-0.34	0.85	1.0	0.62	0.85	-0.26	-0.26	-0.54	-0.33	0.85	-0.29
TNR	0.11	-0.13	1.0	0.41	0.11	-0.49	0.12	-0.42	0.15	-0.52	0.12	-0.13	0.18	0.11	0.03	-0.18	-0.06	-0.24	0.12	-0.24
PPV	0.4	-0.07	0.41	1.0	0.46	-0.06	0.28	-0.7	0.44	-0.07	0.4	-0.07	0.37	0.41	0.06	-0.44	-0.17	-0.53	0.4	-0.03
IOU	0.98	0.84	0.11	0.46	1.0	-0.33	0.89	-0.05	0.99	-0.34	0.98	0.84	0.76	0.98	-0.23	-0.47	-0.61	-0.58	0.98	-0.27
GCE	-0.34	-0.34	-0.49	-0.06	-0.33	1.0	-0.33	-0.06	-0.38	1.0	-0.34	-0.34	-0.17	-0.33	0.06	0.2	0.18	0.27	-0.34	0.47
VS	0.94	0.77	0.12	0.28	0.89	-0.33	1.0	-0.06	0.9	-0.34	0.94	0.77	0.78	0.92	-0.31	-0.37	-0.68	-0.51	0.94	-0.25
RAVD	-0.04	0.4	-0.42	-0.7	-0.05	-0.06	-0.06	1.0	-0.04	-0.04	-0.04	0.4	-0.2	-0.03	-0.11	0.22	0.06	0.34	-0.04	-0.09
MI	0.99	0.85	0.15	0.44	0.99	-0.38	0.9	-0.04	1.0	-0.39	0.99	0.85	0.78	0.99	-0.26	-0.46	-0.64	-0.58	0.99	-0.3
VOI	-0.35	-0.34	-0.52	-0.07	-0.34	1.0	-0.34	-0.04	-0.39	1.0	-0.35	-0.35	-0.2	-0.34	0.08	0.21	0.21	0.29	-0.35	0.47
CKS	1.0	0.85	0.12	0.4	0.98	-0.34	0.94	-0.04	0.99	-0.35	1.0	0.85	0.81	1.0	-0.29	-0.43	-0.68	-0.56	1.0	-0.28
AUC	0.85	1.0	-0.13	-0.07	0.84	-0.34	0.77	0.4	0.85	-0.35	0.85	1.0	0.62	0.85	-0.26	-0.26	-0.54	-0.33	0.85	-0.29
VC	0.82	0.62	0.18	0.37	0.76	-0.17	0.78	-0.2	0.78	-0.2	0.81	0.62	1.0	0.81	-0.41	-0.43	-0.77	-0.62	0.81	-0.15
MCC	1.0	0.85	0.11	0.41	0.98	-0.33	0.92	-0.03	0.99	-0.34	1.0	0.85	0.81	1.0	-0.32	-0.44	-0.7	-0.56	1.0	-0.28
PBD	-0.29	-0.26	0.03	0.06	-0.23	0.06	-0.31	-0.11	-0.26	0.08	-0.29	-0.26	-0.41	-0.32	1.0	0.07	0.84	0.13	-0.29	0.14
HD95	-0.43	-0.26	-0.18	-0.44	-0.47	0.2	-0.37	0.22	-0.46	0.21	-0.43	-0.26	-0.43	-0.44	0.07	1.0	0.31	0.9	-0.43	0.12
MHD	-0.68	-0.54	-0.06	-0.17	-0.61	0.18	-0.68	0.06	-0.64	0.21	-0.68	-0.54	-0.77	-0.7	0.84	0.31	1.0	0.45	-0.68	0.21
ASSD	-0.56	-0.33	-0.24	-0.53	-0.58	0.27	-0.51	0.34	-0.58	0.29	-0.56	-0.33	-0.62	-0.56	0.13	0.9	0.45	1.0	-0.57	0.14
ARI	1.0	0.85	0.12	0.4	0.98	-0.34	0.94	-0.04	0.99	-0.35	1.0	0.85	0.81	1.0	-0.29	-0.43	-0.68	-0.57	1.0	-0.28
OASSD	-0.28	-0.29	-0.24	-0.03	-0.27	0.47	-0.25	-0.09	-0.3	0.47	-0.28	-0.29	-0.15	-0.28	0.14	0.12	0.21	0.14	-0.28	1.0

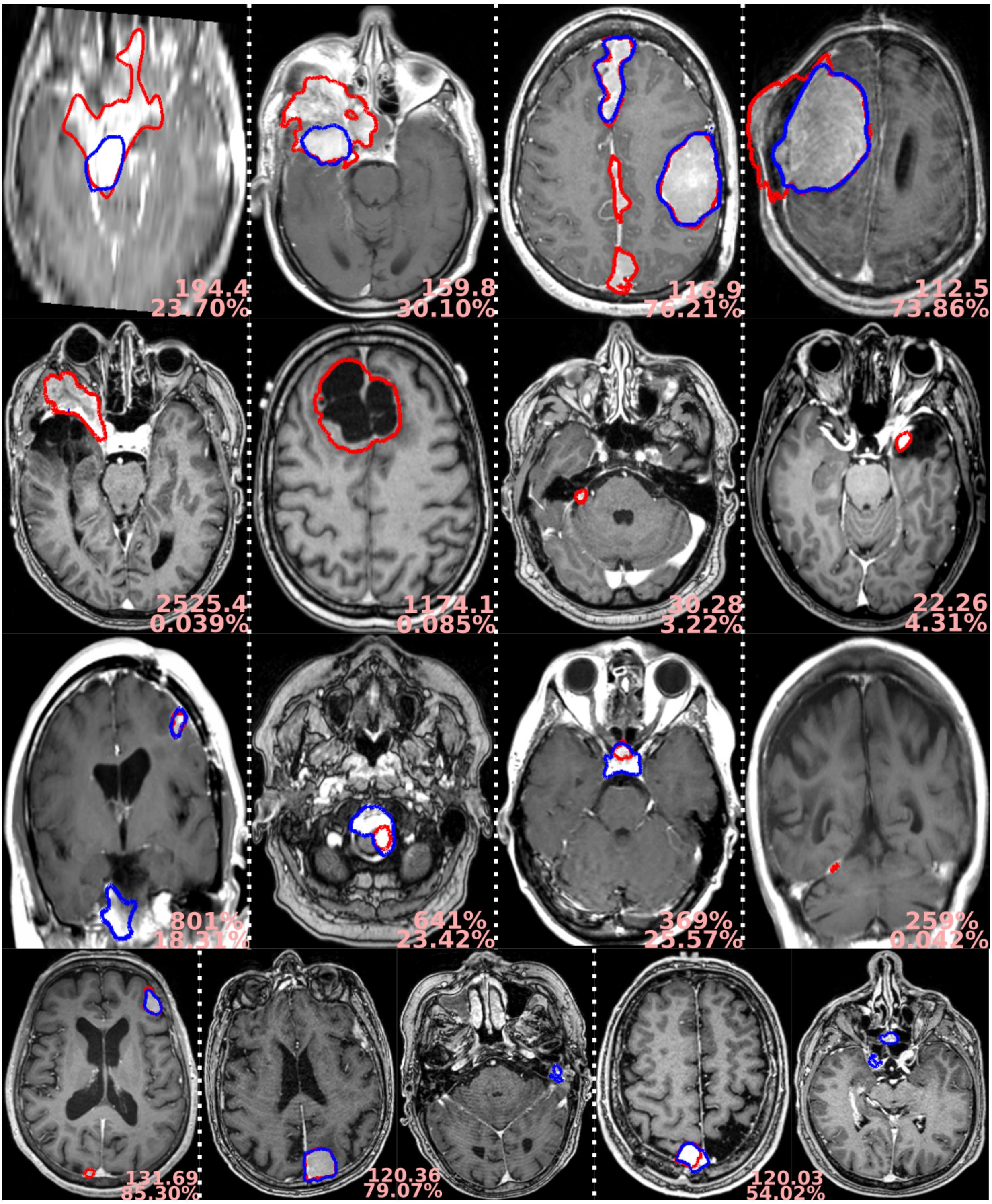

Figure S3. Examples of meningioma outliers where the ground truth is shown in red and the automatic segmentation in blue. Each patient is separated by a dotted white line, and each row focuses on a different metric, from top to bottom: GCE, PBD, RAVD, and HD95. For each patient, both metric of interest and Dice scores are provided in light red.

2.4 Metastases segmentation

Overall segmentation performances for the meningioma tumor type are reported fold-wise in Table S7. The average Dice score is quite stable across the different folds, at 87%, given the almost identical number of samples they hold. The same trend can be noticed for the patient-wise and object-wise metrics across the board.

The correlation matrix between some of the considered validation metrics is shown in Table S8. Visual segmentation results are illustrated in Fig. S4 where the GCE, PBD, RAVD, and HD95 metrics scored orders of magnitude above their average, indicating clear outliers not identifiable by the Dice metric only. Overall, the metastasis model has subpar precision because of the smallest amount of training data, suffering a bit from transfer learning from the HGG model, which can be addressed with more training data.

As can be seen in the second row, the model segmented a cavity in the leftmost example. A large tumor portion has been missed at the top of the head in the second column, maybe as a resulting limitation from performing skull stripping.

Table S7. Fold-wise overall performance for the metastase segmentation.

		Pixel-wise		Patient-wise			Object-wise		
Fold	# Samples	Dice	Dice-TP	F1-score	Recall	Precision	F1-score	Recall	Precision
0	79	87.51 ± 19.98	90.96 ± 10.05	96.74	96.20	97.28	87.87	82.73	93.68
1	80	89.03 ± 16.82	91.31 ± 09.05	98.05	97.50	98.61	89.08	82.81	96.36
2	80	89.63 ± 15.16	89.63 ± 15.16	98.73	100.00	97.50	91.16	86.73	96.08
3	75	84.13 ± 22.26	86.43 ± 17.60	96.87	97.33	96.41	87.94	82.48	94.17
4	80	88.15 ± 19.88	91.58 ± 09.81	97.25	96.25	98.27	87.46	79.22	97.60

Table S8. Metrics correlation matrix for the metastasis segmentation. The color intensity of each cell represents the strength of the correlation, where blue denotes direct correlation and red denotes inverse correlation.

	Overlap						Volume		Information theory		Probabilistic					Spatial distance			Instance-wise	
	Dice	TPR	TNR	PPV	IOU	GCE	VS	RAVD	MI	VOI	CKS	AUC	VC	MCC	PBD	HD95	MHD	ASSD	ARI	OASSD
Dice	1.0	0.89	0.12	0.41	0.99	-0.27	0.96	-0.1	0.99	-0.3	1.0	0.89	0.73	1.0	-0.82	-0.44	-0.74	-0.57	1.0	-0.73
TPR	0.89	1.0	-0.09	-0.01	0.88	-0.16	0.84	0.26	0.87	-0.18	0.89	1.0	0.57	0.88	-0.65	-0.43	-0.51	-0.48	0.89	-0.61
TNR	0.12	-0.09	1.0	0.48	0.15	-0.88	0.09	-0.2	0.18	-0.85	0.12	-0.09	0.12	0.13	-0.04	-0.08	-0.12	-0.16	0.12	-0.25
PPV	0.41	-0.01	0.48	1.0	0.45	-0.36	0.32	-0.66	0.46	-0.35	0.41	-0.01	0.41	0.44	-0.37	-0.2	-0.54	-0.35	0.41	-0.35
IOU	0.99	0.88	0.15	0.45	1.0	-0.3	0.92	-0.06	1.0	-0.32	0.99	0.88	0.68	0.99	-0.73	-0.46	-0.69	-0.57	0.99	-0.73
GCE	-0.27	-0.16	-0.88	-0.36	-0.3	1.0	-0.23	0.05	-0.35	1.0	-0.28	-0.16	-0.21	-0.29	0.14	0.17	0.19	0.29	-0.28	0.44
VS	0.96	0.84	0.09	0.32	0.92	-0.23	1.0	-0.11	0.92	-0.25	0.96	0.84	0.71	0.94	-0.83	-0.39	-0.72	-0.53	0.96	-0.72
RAVD	-0.1	0.26	-0.2	-0.66	-0.06	0.05	-0.11	1.0	-0.09	0.05	-0.1	0.26	-0.44	-0.11	0.35	0.03	0.63	0.21	-0.1	-0.08
MI	0.99	0.87	0.18	0.46	1.0	-0.35	0.92	-0.09	1.0	-0.37	0.99	0.88	0.7	1.0	-0.77	-0.45	-0.72	-0.58	0.99	-0.74
VOI	-0.3	-0.18	-0.85	-0.35	-0.32	1.0	-0.25	0.05	-0.37	1.0	-0.3	-0.18	-0.23	-0.31	0.17	0.17	0.22	0.31	-0.3	0.47
CKS	1.0	0.89	0.12	0.41	0.99	-0.28	0.96	-0.1	0.99	-0.3	1.0	0.89	0.73	1.0	-0.82	-0.44	-0.74	-0.57	1.0	-0.73
AUC	0.89	1.0	-0.09	-0.01	0.88	-0.16	0.84	0.26	0.88	-0.18	0.89	1.0	0.57	0.88	-0.65	-0.43	-0.51	-0.48	0.89	-0.61
VC	0.73	0.57	0.12	0.41	0.68	-0.21	0.71	-0.44	0.7	-0.23	0.73	0.57	1.0	0.73	-0.73	-0.47	-0.88	-0.79	0.73	-0.33
MCC	1.0	0.88	0.13	0.44	0.99	-0.29	0.94	-0.11	1.0	-0.31	1.0	0.88	0.73	1.0	-0.81	-0.45	-0.74	-0.58	1.0	-0.72
PBD	-0.82	-0.65	-0.04	-0.37	-0.73	0.14	-0.83	0.35	-0.77	0.17	-0.82	-0.65	-0.73	-0.81	1.0	0.3	0.81	0.48	-0.82	0.55
HD95	-0.44	-0.43	-0.08	-0.2	-0.46	0.17	-0.39	0.03	-0.45	0.17	-0.44	-0.43	-0.47	-0.45	0.3	1.0	0.43	0.79	-0.44	0.15
MHD	-0.74	-0.51	-0.12	-0.54	-0.69	0.19	-0.72	0.63	-0.72	0.22	-0.74	-0.51	-0.88	-0.74	0.81	0.43	1.0	0.68	-0.74	0.39
ASSD	-0.57	-0.48	-0.16	-0.35	-0.57	0.29	-0.53	0.21	-0.58	0.31	-0.57	-0.48	-0.79	-0.58	0.48	0.79	0.68	1.0	-0.57	0.22
ARI	1.0	0.89	0.12	0.41	0.99	-0.28	0.96	-0.1	0.99	-0.3	1.0	0.89	0.73	1.0	-0.82	-0.44	-0.74	-0.57	1.0	-0.73
OASSD	-0.73	-0.61	-0.25	-0.35	-0.73	0.44	-0.72	-0.08	-0.74	0.47	-0.73	-0.61	-0.33	-0.72	0.55	0.15	0.39	0.22	-0.73	1.0

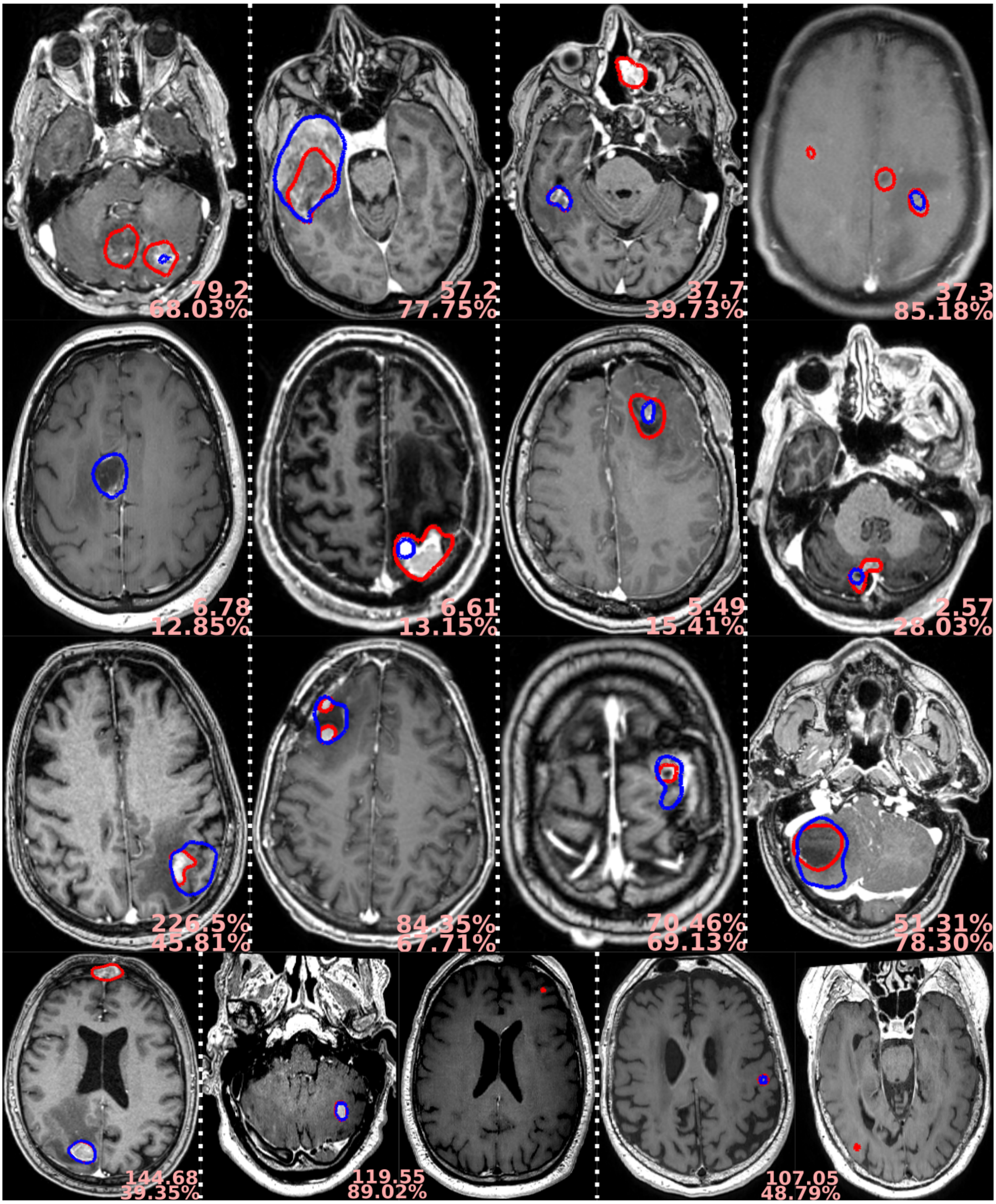

Figure S4. Examples of metastasis outliers where the ground truth is shown in red and the automatic segmentation in blue. Each patient is separated by a dotted white line, and each row focuses on a different metric, from top to bottom: GCE, PBD, RAVD, and HD95. For each patient, both metric of interest and Dice scores are provided in light red.

2.5 Runtime

Using a representative MRI volume of each tumor type considered, the following four tables indicate runtime for the different steps performed during the process to generate the standardized clinical reports. Results are reported when using the high-end computer, either using the stand-alone software solution or the 3D Slicer plugin.

The most noticeable difference comes from the penultimate step (i.e., Apply reg.) going from 183 seconds with the lower grade glioma volume to 792 seconds with the metastasis volume. The size of the MRI volume has a huge impact on the runtime necessary to apply the inverse registration transform on all cortical and subcortical structures atlases, in order to express them in the patient space. For the metastasis tumor type, the other steps performing volume-wise operations (e.g., tumor segmentation or registration) also have a longer runtime given the dimensions of the MRI volume, when compared to a typical glioblastoma MR scan.

Table S9. Runtime distribution per step in the standardized report pipeline, computed and averaged using an high-end computer for the glioblastoma tumor type, each expressed in seconds.

	Brain segm.	Tumor segm.	Reg. preproc	Registration	Apply reg.	Features comp.
Raidionics	25.72 ± 0.123	21.99 ± 0.177	1.394 ± 0.016	37.40 ± 1.41	154.56 ± 0.91	112.55 ± 1.25
Raidionics-Slicer	23.04 ± 00.64	20.63 ± 00.07	1.539 ± 0.109	95.18 ± 0.88	240.13 ± 0.20	120.63 ± 4.05

Table S10. Runtime distribution per step in the standardized report pipeline, computed and averaged using an high-end computer for the lower grade glioma tumor type, each expressed in seconds.

	Brain segm.	Tumor segm.	Reg. preproc	Registration	Apply reg.	Features comp.
Raidionics	18.29 ± 0.402	16.69 ± 0.427	0.778 ± 0.013	32.10 ± 1.32	112.12 ± 3.34	90.28 ± 1.03
Raidionics-Slicer	16.58 ± 0.201	16.24 ± 0.286	0.858 ± 0.032	74.76 ± 1.97	183.41 ± 0.88	89.91 ± 1.92

Table S11. Runtime distribution per step in the standardized report pipeline, computed and averaged using an high-end computer for the meningioma tumor type, each expressed in seconds.

	Brain segm.	Tumor segm.	Reg. preproc	Registration	Apply reg.	Features comp.
Raidionics	19.08 ± 0.445	17.21 ± 0.425	0.865 ± 0.023	37.79 ± 2.63	107.77 ± 1.98	146.08 ± 4.12
Raidionics-Slicer	17.70 ± 0.624	17.07 ± 0.571	0.924 ± 0.016	90.58 ± 1.03	178.92 ± 2.71	144.35 ± 2.49

Table S12. Runtime distribution per step in the standardized report pipeline, computed and averaged using an high-end computer for the metastasis tumor type, each expressed in seconds.

	Brain segm.	Tumor segm.	Reg. preproc	Registration	Apply reg.	Features comp.
Raidionics	68.58 ± 1.454	63.32 ± 1.310	6.906 ± 0.222	72.09 ± 1.12	573.33 ± 9.76	136.96 ± 0.98
Raidionics-Slicer	59.37 ± 1.266	53.85 ± 0.358	7.097 ± 0.174	139.56 ± 3.70	792.76 ± 17.13	135.71 ± 3.71
